# Bovine Milk Fats and Their Replacers in Baked Goods: A Review

**DOI:** 10.3390/foods8090383

**Published:** 2019-09-02

**Authors:** Zhiguang Huang, Letitia Stipkovits, Haotian Zheng, Luca Serventi, Charles S. Brennan

**Affiliations:** 1 Department of Wine, Food and Molecular Biosciences, Faculty of Agriculture and Life Sciences, Lincoln University, Lincoln, Christchurch 7647, New Zealand; 2 Riddet Research Institute, Palmerston North 4442, New Zealand; 3 Dairy Innovation Institute, California Polytechnic State University, San Luis Obispo, CA 93407, USA

**Keywords:** milk lipids, bakery products, fat replacer, shortening, baking activity

## Abstract

Milk fats and related dairy products are multi-functional ingredients in bakeries. Bakeries are critical local industries in Western countries, and milk fats represent the most important dietary lipids in countries such as New Zealand. Milk fats perform many roles in bakery products, including dough strengthening, textural softeners, filling fats, coating lipids, laminating fats, and flavor improvers. This review reports how milk fats interact with the ingredients of main bakery products. It also elaborates on recent studies on how to modulate the quality and digestibility of baked goods by designing a new type of fat mimetic, in order to make calorie- and saturated fat-reduced bakery products. It provides a quick reference for both retailers and industrial manufacturers of milk fat-based bakery products.

## 1. Introduction

Milk contributes approximately one third of human dietary lipid intake [1]. Milk lipids consist of protein and also neutral lipids (triacylglycerols(TAG), monoacylglycerols (MAG), diacylglycerols (DAG), free fatty acids (FFA)) and polar lipids (phospholipids) [2,3]. Milk fats and related dairy products, such as butter, anhydrous milk fats (AMF), cream, cultured milk fats, and cheese (matrix of milk lipids and proteins), have been incorporated into both extruded and baked products, including breads, cakes and biscuits [4,5].

There are several reviews on bread lipids functionalities [6], bakery fat replacers [7], bakery lipids [8], lipid shortenings [9], bakery emulsifiers [10], bread functional ingredients and textural improvers [11], milk lipids in the food system [12], and bread emulsifiers [13]. However, thus far, there has been no review on how milk fats perform their functions in bakery products. Therefore, this review aims to summarize milk fat applications in the bakery industry, and to update results on using milk fats to enhance the quality and nutritional value of baked goods. It also reports on the recent trends in relation to the health concerns of milk fats in baked products, and new ideas to reduce bakery energy density and saturated fatty acids (SFA).

## 2. Structure, Composition and Occurrence

### 2.1. Molecular Structure, Composition and Occurrence

Bovine milk lipids are comprised of 97.5% TAG, 0.36% DAG, 0.027% MAG, 0.027% FFA, and 0.6% phospholipids [14]. There are also some minor lipid classes present in milk, for instance, sterols, carotenoids, lipophilic vitamins, and flavor compounds [2].

The triacylglycerol molecule consists of a glycerol backbone and three fatty acids esterified at the positions of *sn*-1, *sn*-2, and *sn*-3. Two subclasses of phospholipids are glycerophospholipids and sphingolipids. Glycerophospholipids consist of a glycerol moiety with two fatty acids esterified at the positions of *sn*-1 and *sn*-2 and a hydroxyl group at *sn*-3 position, linked to a phosphate group and a hydrophilic residue. The structural details of the hydrophilic residue determine the types of glycerophospholipids, namely phosphatidylcholine (PC), phosphatidylserine (PS), phosphatidylethanolamine (PE), phosphatidylinositol (PI), phosphatidyl-glycerol (PG), and phosphatidic acid (PA) [3]. Sphingolipid consists of sphingosine backbone (ceramide, 2-amino-4-octadecene-1,3-diol), linked to a fatty acid through an amide bond and a polar head. Sphingomyelin (SM) is the predominant subclass of sphingolipids, having a phosphocholine head group. A minor constituent of sphingolipids in milk is glycosphingolipid, of which the polar group is comprised of carbohydrate groups (glucose, galactcose, and lactose) [15].

In intact raw bovine milk, lipids (3.3–4.6% [2]) are present in the form of milk fat globules (MFG), with an average diameter of 0.1–20 μm and are enveloped by a tri-layered phospholipid membrane [16]. The triple-layer membrane consists of a surface-active inner monolayer enveloping TAG in the center and an outer bilayer in contact with the aqueous phase of the milk. The milk fat globule membrane (MFGM) is composed of polar lipids, proteins, glycoproteins, enzymes and minor neutral lipids [17].

### 2.2. Fatty Acid Profile

The most abundant milk fatty acids are palmitic (26.3–30.4%), oleic (28.7–29.8%), stearic (10.1–14.6%), and myristic (8.7–7.9%) acids [14]. Anhydrous milk fats (AMF), known by the US Department of Agriculture (USDA) as 1003, consist of palmitic acid (27.7%), oleic acid (26.5%), stearic acid (12.8%), and myristic acid (10.6%) [2]. Due to a high content of stearic and palmitic fatty acids (melting points at 69.3 °C and 62.9 °C, respectively), milk fats are solid at ambient temperature. Conjugated linoleic acids (CLAs) are isomers of linoleic acids (0.8–2.5%) with the predominant CLAs being *cis*-9 and *trans*-11 CLAs (73–94%) [14].

SFA and monounsaturated fatty acids account for 62.2% and 28.9% (*w*/*w*) of the total fatty acids (FA) in the anhydrous butter oil of United States Department of Agriculture (USDA 1003), respectively, whereas long-chain FAs (LCFAs, C13–C21) accounts for 83.9% of the total FA, compared to medium-chain FA (MCFAs, C6–C12, 8.8%) and short-chain FA (SCFAs, C2–C5, 3.4%) [2]. Unlike LCFAs, SCFAs and MCFAs are absorbed intact as non-esterified fatty acids into the portal bloodstream and metabolized rapidly in the liver [18]. Via gastrointestinal digestion, medium-chain TAG is decomposed into glycerol and MCFAs, which reduces total cholesterol in serum by boosting hepatic synthesis of bile acid [19]. The SFA degrees of main shortening lipids are shown in Table 1 [2]. Lipids of dairy products can be separated by the Folch extraction [20], the Bligh method [21], the Röse–Gottlieb extraction [22], or dichloromethane [23]. Total lipid (TL) content of samples may be measured using gravimetric determination, a Gerber–van Gulik butyrometer, infrared spectrometry in a Milkoscan FT2 apparatus [22], or gas chromatography [24].

### 2.3. Melting Properties and Solid Fat Index (SFI)

**Figure 1:**
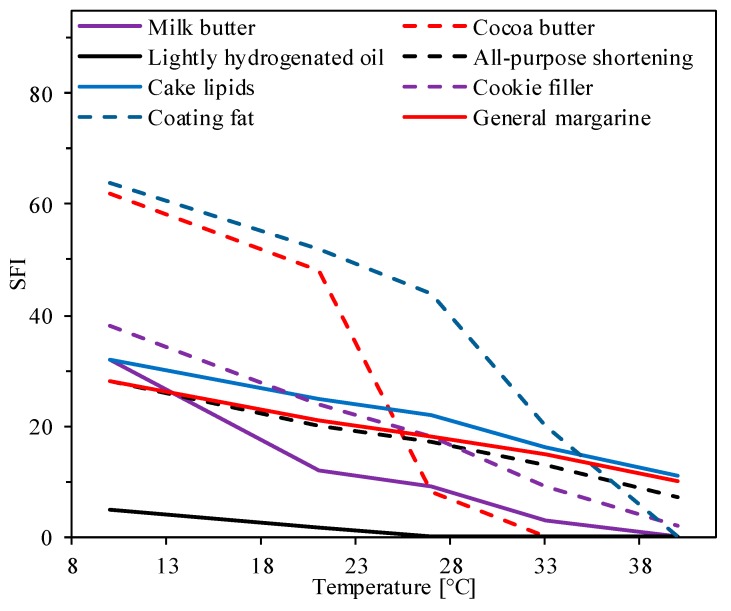
Solid fat index (SFI) profile of typical shortenings for bakery products. Notes: The SFI data was adapted from [25].

The SFI profile of milk fat crystal powder can be measured by pulsed nuclear magnetic resonance (p-NMR) with thermostatic incubation, and differential scanning calorimetry (DSC) can be used to determine the fat melting point [4]. The SFIs of major lipids in bakery products are illustrated in Figure 1 [25]. The SFI profile of milk butter is very similar to that of general use margarine, all-purpose shortening, and cake lipids, and thus, milk butter is interchangeable with other shortenings. Cocoa butter can be used for coating bakery products, whereas milk fats are too soft for coating. Even as a cookie filler, milk fats are not firm enough and need to be formulated with other lipids. To achieve optimum bakery activity, bakery lipids should have 20% SFI at 25 °C and a minimum of 5% SFI at 40 °C [26]. For instance, a blend of stearin fraction of palm-based DAG and palm mid-fraction (50:50 *w*/*w*; SFI at 30% and 10% for 25 °C and 40 °C, respectively; polymorphic form β’ + β; slip melting point 55.4 ± 0.12 °C) makes a better bakery shortening than sunflower oil and palm oil [26]. An SFI profile of less than 15–20% at the dough temperature is too soft to make a shortening. However, fats that are too hard produce adverse effects, for instance, shortening with an SFI of ca. 47.5% at 20 °C produces less acceptable biscuits than shortening with an SFI of ca. 22.5% at 20 °C [27].

### 2.4. Crystalline Polymorphism

Aside from SFI, polymorphic forms of milk fat crystals are also a factor in controlling the bakery activity of lipids [4] Lipids exist in three major polymorphic forms, α, β’, and β, and their stability ascends in the order of α < β’ < β. Lipid crystal α is usually undesirable due to its instability. The β crystal (large plate-like) is stable, but coarse and sandy, whereas β’ form is desired in baked goods since it is fine, needle-shaped, and stable. Enzymatic inter-esterification can rearrange fatty acids on the TAG backbone, creating tightly packed, small β’ crystals, which produce more desirable bakery activity than composite blends [28]. Milk fats, together with other natural edible lipids such as tallow, palm oil, cottonseed oil, and high erucic acid rapeseed oil possess the β’ polymorph, whereas crystals of soybean oil, sunflower oil, coconut oil, palm kernel oil, and lard are usually present in the β polymorph. Crystalline forms may transform into a more stable form as time and temperature change [29]. The crystalline polymorphism can be determined by using an x-ray diffractometer (XRD) [30]. Characteristic peaks of the acylglycerol emulsifier-shortening blend at 4.15 Å and 4.6 Å are from α and β forms, respectively, whereas the β’ form demonstrates three signals at 3.8 Å, 4.2 Å and 4.3 Å. In addition, sub-β and sub-β’ forms may cause peaks at 4.5 Å and 4.0 Å, respectively [31].

## 3. Milk Fats for Bakery Products

### 3.1. Milk Fats and Related Dairy Products

Milk fats and related dairy products include butter, anhydrous milk fat, ghee, and cheese (combination of milk lipids and proteins) [32]. Milk butter is the predominant milk fat product used in the bakery industry, comprised of 81.11% milk lipids and 16.17% moisture, approximately (USDA 1145 in Table 2 [2]). In native milk, the enveloped fat globules are dispersed in the serum [33]. During churning, the membrane is disrupted and those milk fat globules aggregate to form butter, separating out from serum (buttermilk) [34]. Cultured lactic butter is more popular in Europe than in the USA, whereas sweet cream butter is more prevalent in the UK and USA than in other countries [35]. Salted butter (1.6–1.7% salt) has a 4-fold shelf life in refrigeration than unsalted butter due to reduced water activity [32]. Ghee is clarified milk fats from butter or cream, with an enriched flavor [36]. A milk butter blend with vegetable oil (e.g., corn oil, canola oil) reduces the overall SFA. Being hydrogenated from vegetable oils (e.g., soybean and palm oil) or animal fats (e.g., beef tallow) to raise the SFI and melting point, margarine is a cheaper substitute for milk butter in the bakery industry [37]. To avoid TFAs resulted from hydrogenation, inter-esterification of vegetable oils (soybean oil, palm stearin, coconut stearin; 20:50:30, *w*/*w*/*w*) by Lipozyme RM IM produces optimized SFI and crystals of β’ polymorphic form, which are equally as effective as commercial margarines [38].

AMF contains 99.48% lipids and 0.24% moisture, respectively (Table 2 [2]). According to the Codex/CFR Alimentary, AMF and butter oil must be comprised of no less than 99.8% and 99.6% lipids, respectively, without additives [32]. The AMF is produced by vacuum drying and removal of nonfat solids from pasteurized cream. First, cream (40% lipids) is concentrated to 70–80% milk fats, and after phase inversion, the milk fats are further dried to no more than 0.1% moisture [32]. The AMF can be produced from both butter and cream [33], and butter oil is made out of butter [32]. For cost-saving, substitution of 30% AMF by hydrogenated vegetable oils (e.g., soya or coconut oil) has been formulated into the shortening of bakery products in Asian countries such as Japan [25]. AMF has a broad melting and crystallization range, fully crystalizing at −40 °C and completely melting at 38–40 °C. Thus AMF can be fractionated into low (<10 °C), middle (10–20 °C), high (>20 °C), and very high (>50 °C for confectionery) melting fractions [39].

Cheese is produced from milk by inoculation with bacteria and separation of the resulting semi-solid curd (33–55% lipids for origin cream cheese, 0.5–16.5% lipids for reduced-fat cheese) from the liquid whey, leading to less-perishable products than milk [40]. Among the most commonly used bakery flavors, cheddar and parmesan cheeses have been used to impart flavor in biscuits or crackers [41]. Aside from flavor enrichment, cheese can also be used as a bakery coating or filling lipids [42]. A typical cheddar cheese contains 33.31% lipids (Table 2 [2]). Gas chromatography analysis of enzymatically-modified white cheese for bakery flavor revealed 58 volatile compounds of seven chemical classes including alcohols (12), aldehydes (8), ketones (10), esters (8), acids (11) and hydrocarbons (9), among which most compounds were produced by metabolism of carbohydrates, milk fats and amino acids [42]. Kefir cheese culture fermentation yielded volatile compounds, for instance, diacetyl (i.e., major buttery aroma), acetaldehyde, ethanol, and acetone by metabolism of probiotic bacteria (e.g., lactic acid bacteria, *L. acidophilus*, *Bifidobacterium* spp.) and yeasts (e.g., *Saccharomyces* spp. and *Kluyveromyces* spp.) [43]. Bacterial metabolism produces diacetyl, for instance, by *Lactococcus lactis* subsp. [44].

Sour cream, a critical bakery flavor improver, is produced by the moderate-temperature fermentation of cream, and it can also be made by the treatment of acid-producing bacterial cultures on pasteurized cream. Compared to cream, sour cream (typical lipid content 19.35% in Table 2 [2]) is thicker and more acidic, with a longer shelf-life [45].

Furthermore, milk fats are often consumed together with biscuits and breads (e.g., as fillers) [46]. Milk fat products such as butter and AMF are sometimes manufactured as flaked or powdered forms by spray chilling or spray drying, which are easy to disperse [29]. The typical composition of milk fats and related dairy products are illustrated in Table 2 [2].

### 3.2. Functional Roles of Milk Fats in Bakery Products: Baking Activity

Milk fats have been used to perform multifunctional roles in bakery products, for instance, as mouthfeel and flavor improvers, texture improvers, dough conditioners, and anti-staling agents [29]. In addition, milk fats can fulfil a wide variety of functions such as laminating and filling fats, coating or topping lipids, spray oil, and imparting flavor [35]. The functions of milk fats are dependent on the dose and the type of baked products. For instance, they play more strengthening roles in yeast-leavened bread dough than in cookie/biscuit dough or cake batter, whereas cake fats are highly attributed for aeration and whipping in batter agitation [47], and biscuit or cookie laminating fats are mainly responsible for crisping and puffy effects by textural improvement [47]. Almost half of the lipids in coconut oil (USDA 4047) are comprised of lauric acid (41.84%, T_m_ = 43.2 °C), but bovine milk fats (USDA 1003) contain only 2.79% lauric acid in comparison [2]. Therefore, milk butter needs to be blended with cocoa or equivalent to make a bakery coating.

Bakery shortening is defined as the ability of a fat to lubricate, weaken, or shorten the structure of bakery products, thereby providing tenderization effects and other desirable textural properties to bakery products [48]. During the mixing process of dough or batter, lipids interact with gluten and starch particles to strengthen their network, thus improve the gas retention of dough. Hence, bakery products become softened, resulting in consistent grain, lubricated mouthfeel, enhanced heat transfer, and extended shelf life [9]. Shortening lipids are made from milk butter, animal fats (e.g., tallow, lard), or hydrogenated plant oils (e.g., palm oil) [47]. In contrast to standard shortening, lipids such as hydrogenated vegetable fats may be used to replace milk fats for bakery products, such as biscuits [49].

Laminated dough shortening has an SFI of 10–40% for the temperature range of 33 °C to 10 °C, causing a puffy texture for croissants, danishes, and pastries. Milk butter is a benchmark laminated dough preparation agent for appropriate SFI profile and β’-form crystal. Cheap alternatives include hydrogenated shortenings and inter-esterified fats, which lead to a trans fatty acids (TFA) issue or less acceptable sensory quality [4].

Bakery lipids have their characteristic SFI profile, plasticity (processability), and antioxidant stability [50]. For instance, a coconut oil cookie filler is designed as a 59% SFI at 10 °C, 29% at 21.1 °C, and 0% at 26.7 °C onwards, with a melting point of 24.5 °C. In contrast, croissant shortening melts at 39 °C, with an SFI profile of 39% at 10 °C, 27% at 21.1 °C, 22% at 26.7 °C, 19% at 33.3 °C, and 18% at 43.3 °C [47]. Milk fat flavors have been attributed to volatile molecules, including branched-chain fatty acids, lactones, methyl ketones, aldehydes, and other minor compounds, which are originated from milk fats or produced during fermentation, lipolysis, or processing. Milk fat products, such as cheese (e.g., cheddar and feta), cream, sour cream, and butter are all used to improve the sensory properties of bakery products [43].

### 3.3. Interaction of Milk Fats with Other Bakery Ingredients

#### 3.3.1. Lipid–Protein/Starch Interaction

Lipid–protein binding interactions can increase gluten polymerization. However, the ionic amphiphilic binding will cause interface aggregation due to charge neutralization, and therefore, this interaction may also decrease surface activity as the lipid concentration at the aqueous–oil interface increases to a certain level, which leads to the disruption of protein–protein interactions in the interfacial film [51]. The gluten–lipid interaction yields a dynamic balance of surface activity, altering the surface activity and aeration ability. This mechanism is critical for dough rheological characteristics and product textural properties. Horra et al. [52] compared refined milk fats (SFI 38% at 25 °C) and margarine shortening (SFI 5–25% at 25 °C) and found, through confocal microscopy, that the gluten network with milk fats is less developed and more orderly structured (with isolated starch particles) than the network formed with margarine shortening, thereby producing greater elastic and viscous moduli, and higher puff pastry.

During dough mixing, milk fats coat the gluten network and starch particles, reducing the water hydration capacity of the dough [6]. With the formation of an extensible gluten film by hybrid hydration and lipid coating, the lipid crystals decrease the surface tension of the gluten film (lubrication effects), promoting aeration of the dough [9]. The crystals align their orientation along the air cells and stabilize them. Milk fats (β’ polymorph) aerate more effectively than soybean oil (β polymorph) by forming fine and consistent gas bubbles [53].

During dough fermentation and proofing, the fat crystals further melt and become absorbed at the gas–liquid interface with elevated temperatures. They re-orientate along the interface plane and hold the yeast-leavened carbon dioxide in the gas cells [54]. Low melting fats or oils have been found to be much less effective in gas retention at this stage [55].

During baking, starch particles become gelatinized and the gluten film turns into a permanent cross-linked thin film together with lipids, with the crust drying and browning (the Maillard reaction) taking place concomitantly [56]. Without lipids, the bubbles tend to coalesce or collapse and produce coarse crumb grain, whereas shortening fats lead to fine crumb grain and consistent porosity [57]. Hydrogenated fats produce a stronger dough and more tender cookies than sunflower oil [58].

When the baked products cool down, amylose and amylopectin crystallize and retrograde in the early stages of storage and over the course of shelf life, respectively [59]. Using low-frequency NMR, it has been found that water migrates from crumb to crust or to amylopectin during storage, and immobilization of moisture will reduce water activity and decrease crystallization of amylopectin, thereby inhibiting the staling rate [60]. Most bread staling mechanisms can be explained by water migration and therefore reducing water activity by changing the gluten network and reducing hydrophilicity of the bread crumb can slow down the rate of bread crumb staling. During bread storage, starch polymer retrogrades concomitantly with fat re-crystallization [61]. This concurrent polymorphic conversion from β’ to β has been evidenced by powder XRD analysis for croissant samples [62].

In brief, shortenings such as milk fats have effects on the lubrication/stabilization (mixing), gas retention (proofing), textural tenderization (baking), and anti-staling (storage) properties of bakery products. The addition of emulsifiers can consolidate the above effects, thus reducing the amount of shortening lipids required. The interaction of lipids with other ingredients is different among breads, cookies, biscuits and cakes, such as with the additional interaction of lipids with egg components.

#### 3.3.2. Starch–Lipid Complexes

Starch can form complexes effectively with MAG, as it can with fatty acids, but TAG does not form complexes with starch [63]. A previous report has shown that four kinds of lipids: monopalmitate glycerol (96.3%), (palmitic acid (41.8%), dipalmitate glycerol (DPG, 1.1%), and tripalmitate glycerol (8.3%) have reduced complexing ability [63]. The starch–lipid complexes have been found to lower the glycemic load of bakery products and impact on their staling processing. Using confocal laser scanning microscope (CLSM) and scanning electron microscopy (SEM), both non-inclusion and inclusion lotus seed starch–lipid complexes have been identified, causing slow digestibility of starch [64]. The complexing index of debranched starch–stearic acid complexes reached 89.31% [65], while that of the native starch (yam)–palmitic acid complexes (2%, *w*/*w*, starch base) was maximized as 26.39% [66]. In addition to the reduction of the starch glycemic index, high amylose corn starch–lipid complexes inhibited the staling process of baked goods [67], as also evidenced by a recent report, where the firmness of wheat bread during storage was significantly reduced by resistant starch [68].

#### 3.3.3. Emulsifier Functionalities during Dough/Batter Forming

Emulsifiers can be used to disperse milk fats, enhance their baking activity, assist ingredient blending and emulsification during dough/batter formation, and promote aeration and air distribution, especially for cake batter [29]. Commonly used emulsifiers for baked goods include MAG and DAG (E471), lecithin (E322), sodium stearoyl lactylate (SSL, E481), and diacetyl tartaric acid ester of mono- and diacylglycerols (DATEM, E472e) [11]. For instance, in a high-ratio layer cake, 5% MAG was formulated into the shortening [31].

Similar to the baking activity of shortening, anionic emulsifiers such as DATEM, SSL, and calcium stearoyl-2-lactylate (CSL) are useful in both dough strengthening and bread softening, as are the nonionic emulsifiers (sucrose esters of fatty acids (SE), polysorbate-60 (poly-60)). Lecithin and distilled MAG have no strengthening effects [13]. Fu et al. [31] compared distilled MAG and four acylglycerols (40%) of octanoic acid (8:0), palmitic acid (16:0), stearic acid (18:0), and linoleic acid (18:2) and found that monopalmitate glycerol and monostearin glycerol led to a higher SFI and finer crystals (β’ form), thus increasing aeration ability in batter formation and tenderizing the crumb of layer cakes. In contrast, monooctanoic glycerol and linoleic acid glycerol produced adverse effects to the SFI and β’ form crystals, thereby reducing cake size and increasing its firmness. In addition, lecithin and distilled monostearate stabilized the shortening crystals and increased the air-absorbing ability on both beef tallow and hydrogenated palm oil [69]. Using digital imaging of crumb micro-structure, the emulsifier functionality in assisting air aeration was recognized. At the same level of dough hydration, five emulsifiers (DATEM, SSL, distilled MAG, lecithin, and polyglycerol esters of fatty acids (PGEF)) increased the bread dough permeability and gas retention ability, resulting in increased gas bubble number and homogeneity [70].

## 4. Milk Fats for Bakery Products

Breads use less fats and sugar than biscuits and cakes, and biscuit recipes use less water than breads and cakes. For instance, breads (AACC 10–10 recipe, flour based) are comprised of wheat flour, 6% sugar, 5% milk butter, 1.5% salt, 1.5% yeast, and 60% water; biscuits (AACC 10–54) consist of wheat flour; 42% sugar, 40% shortening, 1% skim milk powder, 1.25% salt, 1% sodium bicarbonate, 0.5% ammonium bicarbonate, 1.5% high fructose corn syrup, 22% water; high-ratio cakes (AACC 10–90) are made out of wheat flour, 140% sugar, 50% shortening, 2% emulsifier, 12% dry skimmed milk powder, 5.5% baking powder, 9% egg white powder, 3% salt, and ca. 135% water [71]. Cakes are distinct from other products for containing egg, though some other products may also contain egg.

### 4.1. Bread Fats

Milk fats account for 3–4% of a bread recipe [71]. During dough mixing, both starch particles and gluten become hydrated, and the gluten proteins polymerize through reactions between the sulfhydryl (–SH) groups and disulfide (–SS–) bonds, forming an extensive, interlinked dough skeleton [72]. Milk fats mainly perform three kinds of functions in bread dough. First, lipid crystals brace the developed gluten network as a plasticizer. In this instance, shortening oil (e.g., sunflower oil) exhibits far less of an effect on the developing strong gluten network than shortening fats and milk butter due to less SFI [58]. Secondly, lipid crystals align themselves to the gas–liquid interface of bubbles during dough mixing, exerting lubricating effects [73]. Lastly, the lipid crystals enhance the stability index of bread dough [74], and the β’ crystal-stabilized bubbles are larger than that of β crystals [75]. β’ crystal lipids aerate dough more effectively than β crystal lipids [76].

During dough fermentation at 40 °C, yeast digests glucose and emits carbon dioxide and ethanol. Newly produced carbon dioxide diffuses into gas bubbles and leavens dough to 1–1.5-fold in height [77]. Milk fats will then melt and form an extensible thin film, further stabilizing the gas bubbles [78], whereas doughs with insufficient lipids will leak gas via the gluten network due to the penetration or rupturing of the cell wall [78]. However, a high concentration of lipids will inhibit dough rising, as aggregated gluten and solid lipid crystals exert low elasticity, thereby hindering the expansion of bubbles [63].

Upon heating, the cells expand with carbon dioxide diffusion and moisture/ethanol evaporation. With the moisture mobilization and heat transfer, gluten and gelatinized starch become solidified and form a fine crumb texture, while at the same time, the bread crust dries and turns brown due to the Maillard reaction [73]. Shortening fats melt fully and form an elastic thin film together with gluten along cell walls, again stabilizing gas cells [54]. Solid fat-incorporated breads exhibit increased porosity, loaf volume, and softness [79]. During baking, with the melting and gelatinization of starch particles, fat globules melt and form gas cells. The dough moisture migrates towards the edges of gas bubbles to evaporate. Eventually, the bread forms an interlinked porous texture [80].

During the staling process, the bread crust becomes leathery and the crumb turns rigid and unresilient, in parallel to the losses of aroma and eating quality [81]. The migration of moisture across the crumb and crust leads to elevated bread rigidity. In addition, amylose and amylopectin retrograde successively over shelf life [56].

There are several approaches to delay the staling of bread, for instance, by the addition of plasticizers, cross-linkers and fillers, or by the modulation storage temperature to inhibit deformation during staling [61]. Milk fats have sufficient SFI at ambient temperature, and thus, they are able to act as plasticizers to increase storage stability, as well as change the thermoplastic properties.

### 4.2. Biscuit Fats

Biscuits are among the most consumed bakery products worldwide, and they are formulated with flour, fat, sugar, milk, water, eggs (optional), and salt into a viscous dough, and are baked on a flat surface [71]. In addition to lubrication and aeration in dough forming, biscuit fats perform such roles including filling, laminating, coating, surface spray, nutritional value, sensory, and tenderization. Fats (T_m_ ca. 33 °C to give smooth mouthfeel, SFI 53% at 20 °C and 3% at 35 °C) constitute around half of the biscuit filler, in which inappropriate melting points will cause brittleness or filling collapse. Coating fats are usually cocoa butter equivalents (T_m_ ca. 36.6 °C), whereas typical spray lipids approximately possess SFI profiles of 22% at 20 °C and 0.5% at 35 °C [29]. Milk fats can be formulated compositely to fulfill these roles. Milk butter (no less than 7%, flour based) and cheese have been used to make premium butter biscuits (USDA 18214 [2]) and cheese crackers (USDA 45080543 [2]), imparting a buttery aroma. Moreover, cookies also utilize milk butter powder to laminate the dough sheets into several discrete layers, creating a puffy effect on the end product [29]. Enzymatically hydrolyzed or cultured milk fats have been used as flavor agents [42,44]. Aside from the above functionalities, milk butter also serves as a nutritional ingredient. For instance, AMF and butter is comprised of polyunsaturated fats and lipophilic compounds such as vitamin E and β-carotene [35]. In addition, milk fats are natural lipids, without the trans-fatty acid issues of other vegetables fats such as hydrogenated shortenings [82].

Crackers are usually salty biscuits, based on layered dough, whereas cookies are normally made out of high fat and sugar recipes (short-dough [83], more cake-like). To counterbalance gluten development with syrup, comparable fats are added to confine starch granule swelling and limit dough forming [29]. Cookie dough is short-formed, and therefore a chemical leavening agent is used to increase its volume. The lipid content of leavened cookies and crackers is 7–20%, whereas unleavened cookies can have a lipid content as high as 16–33% (dough-based, Table 3). A typical cracker recipe incorporates 23.1% of milk butter (flour-based) [35]. In contrast to breads (35–45% moisture), the moisture content of cookies and biscuits are comparably low. For instance, crackers and cookies in contain 2.75% and 5.9% moisture, respectively [27], and thus they can sustain a long shelf life. Compared to cookies, cracker recipes have no sugar (Table 3).

### 4.3. Milk Fats in Cakes

Cake batter is an emulsion of flour, sugar, shortening, egg, and other minor ingredients [88], and cakes contain more lipids and sugar than breads. Compared to biscuits, milk fats, especially butter, play a greater role in cakes than in biscuits. Yellow cakes (Table 4) use butter and whole egg, resulting in a rich color, tender grain, and milky flavor. White cakes, on the other hand, usually use egg white and shortening instead of milk butter (Table 4). Pound cakes require equal amounts of flour, whole egg, milk butter, and sugar (Table 4), leavened by baking powder. Distinctly, butter may be absent in sponge cake recipes, where egg performs the aeration function in batters, creating foam and an airy grain.

In a layer cake formula ([89], Table 4), the amount of sugar is not greater than the quantity of wheat flour (both 100 g), and the egg amount (49.1 g) is equal or comparable to the amount of shortening (40.91 g). The amount of liquid in the recipe (114.89 g, Table 4) may be equal to or greater than the amount of sugar in the recipe (100 g, Table 4) [90]. Conversely, high-ratio cakes use more sugar than wheat flour (Table 4). To counterbalance the inhibitory effect of excess sugar on starch gelatinization [90], an extra amount of egg is added to strengthen the formula (60 g egg vs. 40 g shortening [31] in Table 4).

Cake fats perform similar functions to bread lipids, for example, air incorporation, air cell stabilization, structure tenderization, and elevation of oven spring [91]. Propylene glycol monostearate (PGMS, 1.8% *w*/*w*), glycerol monostearate (GMS, 1% *w*/*w*), and lecithin (0.8% *w*/*w*) blended with soy bean oil are equally as effective as commercial liquid shortenings in increasing cake size and softness. However, liquid shortening cakes exhibit a reduced firming rate compared to cakes containing plastic shortening, seen over the course of three-weeks in storage [91].

## 5. Milk Fats Replacement

To make cost effective bakery products, these milk fats need to be replaced with more economic sources. In addition, milk fats are high-calorie (3665 kJ/100 g for AMF in Table 2), highly-saturated lipids (ca. 66% in Table 1). Table 5 illustrates some fat replacers used in bakery products. The high content of shortenings in biscuits and cakes, together with the total lipid content in some bakery products (1812 kJ/100 g in Table 6), catalogues them as high-calorie foods (>1675 kJ/100 g [95]), as shown in Table 6. In order to produce bakery goods that are low in calories and saturated lipids, milk fats need to be substituted. There has been interest in using resistant starch (RS) emulsions to substitute bakery fats by 25–50%. In this regard, four forms of starch (2.6–46% RS) exhibited great potential in improving cookie/cake size and symmetry due to the extra hydration capacity of the added starches, while maintaining color and sensory score [96]. Specialty fats (e.g., hydrogenated fats) in the bakery industry have been used to improve texture, shelf life and sensory acceptance. However, they are associated with high serum levels of low density lipoprotein and cholesterol, and the subsequent development of atherosclerosis [30]. Oleogels are recent alternatives to reduce SFA, as illustrated in Table 5. It has been found that fat replacement has less impact on the acceptability of biscuits than sugar reduction [97].

In contrast to the moisture-retention and staling-retardation effects of carbohydrate-based replacers, protein-based replacers perform functions as texturizers. For instance, milk whey protein concentrate has been compositely used to substitute fats [113]. In addition, enzymes can also reduce shortening use, by targeting the endogenous flour lipids. Fugal lipase, e.g., Lipopan F, has been successfully developed to hydrolyze flour lipids to replace milk fats [79,114,115]. In another report, amylase-hydrolyzed starch was used to replace shortening, and achieved a comparable loaf size and consistency, but lower springiness and softness [116]. In general, reduced-fat bakery products have shown poorer performance in regards to mouthfeel, flavor, and texture properties than standard bakery products [117].

### 5.1. Carbohydrate-Based Milk Fat Mimetics

Carbohydrate-based fat mimetics are the most common milk fat replacers, including plant polysaccharides, dietary fiber, and starch [118]. These fat mimetics have been initially designed to generate sufficient baking activity, such as moisture retention, texturizing, and mouthfeel, whereas yielding only half to a quarter of the total calories of fats. However, in terms of flavor, palatability, crumb consistency, appearance, and customer acceptances, these replacers are less effective than milk fats. Recently, dietary fiber (e.g., inulin [119] and pectin [120]) and other resistant starches (e.g., Emjel) have been added to cookie and cake recipes [96], and they achieved similar textural properties to full-fat bakery products. Pectin (Yuja pomace) gel substitution (10%, *w*/*w*) led to the same level of volume and textural properties as shortening cake (AACC 10–90), with elevated softness and whiteness [120]. Inulin (e.g., *Agave angustifolia* fructans) replacement (20%) led to similar sensory properties and enhanced prebiotic activity [121]. Light microscopy images showed that, with the replacement of shortening fats with β-glucans from an edible mushroom in the batter recipe, the population of gas bubbles became decreased, with broader size distribution, which indicated the loss of stabilization by forming an interfacial lipid film along bubbles during batter forming [122].

### 5.2. Lipid-Based Milk Fat Mimetics

Unsaturated lipids or low-calorie lipids have been used to replace milk fats. For instance, replacement of butter in breads by rapeseeds caused a 91% reduction of low-density-lipoprotein-cholesterol in plasma [123]. Margarine is a cheap alternative to milk fats. However, the high water content of margarine limits its use in biscuit manufacturing. Animal fats have been used to inter-esterify with plant oils (e.g., canola oil) to prepare bread shortenings [124], and cookies prepared with oils were firmer than full-fat cookies [125], whereas shortening (palm oil) and emulsifiers together have produced cakes with a similar firmness to cakes prepared with fats [91]. Inter-esterified beef tallow caused slower crystallization than tallow, and brought about an SFI increment of approximately 11% and 5% at 25 °C and 40 °C, respectively, thus increasing cake size and textural consistency. Inter-esterification of the beef tallow-palm medium fraction produced similar plasticity and operability of shortening to beef tallow [126]. The addition of MAG and tripalmitin induced the formation of a polymorphic β-form, accelerating the processing of crystal formation and reducing the size of crystals [127].

### 5.3. Emulsion-Based Milk Fat Mimetics

Oleogels have been fabricated to structure vegetable oils for bakery products, in order to reduce SFA and trans-fatty acids from the diet, as illustrated in Table 5. Oleogels were fabricated by thermal dispersion of sunflower oil into SSL (7–13%, *w*/*w*) at 75 °C [30]. Candelilla wax–canola oil oleogels reduced cookie SFA to ca. 8%, without damaging eating quality [100]. In another study, beeswax–sunflower oil oleogels reduced SFA in cakes to 14–17% from 58% in full-fat cakes [98]. In a previous report, monoacylglycerol organogels and sunflower oil-loaded hydrogels were used to replace shortening fats (palm oil), by 81% [128]. MAG–sunflower/palm oil (0.5%/7%, flour based) water gels have been formulated into bread recipes (4.7% MAG, 55.8% oil, and 39.5% water, *w*/*w*/*w*) [129]. Edible oleogels enhanced nutritional profiles and bioactive benefits [130], and showed important features, such as thermo-reversibility and thixotropy [131]. SSL (7%) has been used as a gelling agent to structure sunflower oil oleogels, creating a crystal network similar to that of TAG [30].

Gels of hydroxypropyl methylcellulose (HPMC)/sunflower oil produced more acceptable biscuits than milk fat, vegetable shortening, sunflower oil/xanthan gum, olive oil/HPMC, and olive oil/xanthan gum [132]. A 15% replacement of HMPC/inulin made crisper biscuits than full-fat shortening [133]. Biscuit dough formulated with an HPMC emulsion showed similar rheological properties to dough made out of shortening fats [134].

### 5.4. Whole Foods or Combined Ingredients to Replace Bakery Lipids

Whole foods, such as avocado, chia, and banana, have been used to replace bakery lipids. For instance, chia (*Salvia hispanica L*. oil content 30–40%, protein content 15–25%) is comprised of rich polyunsaturated fatty acids, such as ω-3 fatty acids (linolenic acid, 54–67%) and ω-6 (linoleic acid, 12–21%). A chia mucilage gel (25%) has been shown to be a feasible alternative for pound cake shortening [109]. The use of oatrim (100% ), bean puree (75%) or green pea puree (75%) as fat replacers in biscuits have proven to be equally effective, and avocado puree can replace half of the shortening in both cakes and biscuits [7]. Okra gum from an edible green fruit (flowing plant of the mallow family) has been identified as a fat replacer for reduced-calorie bakery products, improving the nutritional quality of baked goods [135]. Avocado purée as a full replacement of shortening fats has brought about an increase in MUFA by 16.51%. Substitution by half demonstrated comparable acceptability, whereas further fortification with avocado purée caused undesirable flavor and aftertaste, according to the tested panelists in the study [136].

## 6. Conclusions

This review verifies the relevance and significance of milk fats in bakery products. Their roles include altering structural, rheological, nutritional, and sensory characteristics. The milk fats can be used for dough strengthening in bread making, texture softeners in cakes, and sensory improvers in butter biscuits. In addition, they can be used as cookie fillers, laminating fats, topping and coating fats in bakery products. The interaction of milk fats with flour gluten and starch particles provides dough strengthening and texture improving effects to bakery products. Appropriate fat substitution with the design of new matrices such as oleogels and inulin gels can improve the nutritional value of bakery products by reducing the saturated fatty acid content and energy density, and by increasing the nutrient quality, without adversely affecting the textural and sensory properties. In addition, lipase treatment of flour lipids or milk fats can generate emulsifiers including monoacylglycerols, which may enhance the shortening effect of milk fats and thereby reduce shortening use. Milk fatty acid–wheat starch complexes may also be facilitated so as to reduce glycemic response and increase the shelf-life of baked goods.

In conclusion, milk fats have performed multi-functions in both technical importance and nutritional values, especially for high-end, valued-added baked goods. With partial replacement of milk fats in bakery products to balance their saturated lipids, both nutritional quality and customer acceptability can be further improved.

## Figures and Tables

**Table 1:** Composition of shortening lipids.

USDA Code	Shortenings	Total Lipids (g)	SFA (g)	MUFA (g)	PUFA (g)	TFA (g)	SFA:UFA
4582	Canola oil	100.00	7.37	63.28	28.14	0.40	0.08
4506	Sunflower oil	100.00	10.30	19.50	65.70	-	0.12
4669	Soybean oil	100.00	15.25	22.73	57.33	0.68	0.19
4585	Margarine	80.32	14.20	30.29	24.17	14.95	0.26
4037	Rice bran oil	100.00	19.70	39.30	35.00	-	0.27
4615	Composite shortening	99.97	24.98	41.19	28.10	13.16	0.36
4002	Lard	100.00	39.20	45.10	11.20	-	0.70
1056	Cultured sour cream	19.35	10.14	4.59	0.80	0.80	1.88
1145	Butter	81.11	50.49	23.43	3.01	-	1.91
1003	Anhydrous butter oil	99.48	61.92	28.73	3.69	-	1.91
1017	Cheese cream	34.44	20.21	8.91	1.48	1.17	1.95
4513	Palm kernel oil	100.00	81.50	11.40	1.60	-	6.27
4663	Hydrogenated palm kernel oil (filling fat)	100.00	88.21	5.71	-	4.66	15.46
4701	Fully hydrogenated soy oil	100.00	93.97	1.34	0.38	1.15	54.50

Notes: Saturated fatty acids (SFA), mono-, poly-unsaturated fatty acids (MUFA/PUFA), and trans- fatty acids (TFA) of shortening lipids per 100 g adapted from US Department of Agriculture (USDA) Database v.3.9.5.3 [2].

**Table 2:** Proximate nutritional information of main milk lipid products.

USDA Code	Milk Fats	Water (g)	Energy (kJ)	Protein (g)	Lipids (g)	Ash (g)	Carbohydrate (g)
1017	Cheese	52.62	1466	6.15	34.44	1.27	5.52
1005	Cheese	41.11	1553	23.24	29.68	1.85	2.79
1009	Cheddar cheese	36.37	1684	22.87	33.31	3.71	3.37
1053	Cream	57.71	1424	2.84	36.08	0.53	2.84
1056	Cultured sour cream	73.07	830	2.44	19.35	0.51	4.63
1145	Butter	16.17	2999	0.85	81.11	0.09	0.06
1003	Anhydrous butter oil	0.24	3665	0.28	99.48	-	-

Notes: The nutritional data was adapted from US Department of Agriculture (USDA) Database v.3.9.5.3 [2].

**Table 3:** Biscuit recipes based on 100 flour.

Ingredients (g)	Cracker 1	Biscuit 1	Biscuit 2	Biscuit 3	Biscuit 4
Wheat flour	100.00	100.00	100.00	100.00	100.00
Water	27.50	35.71	13.33	20.00	20.00
Shortening	10.50	13.84	44.89	39.90	66.00
Baking Powder	0.80	0.98	1.11	0.50	-
Salt	1.00	0.66	0.93	0.71	2.40
Emulsifier	2.75	0.59	5.00	0.51	1.00
Sugar	-	26.79	60.00	40.40	33.00
Shortening Dough Base	7%	8%	20%	20%	33%
Reference	[84]	[85]	[86]	[83]	[87]

**Table 4:** Cake batter recipes based on 100 g wheat flour.

Ingredients (g)	White Cake *	Yellow Layer Cake	High Ratio Cake	Pound Cake	Sponge Cake	Sponge Cake
Wheat Flour	100.00	100.00	100.00	100.00	100.00	100.00
Granulated Sugar	136.00	100.00	120.00	100.00	81.82	100.00
Water	106.00	65.79	75.00	16.33	-	-
Fresh Egg	60.00	49.10	60.00	100.00	127.27	100.00
Butter or Equivalent	25.00	40.91	40.00	83.55	86.36	100.00
Emulsifier	0.30	-	2.00	0.12	-	-
Skimmed Milk Powder	9.00	8.18	7.00	-	6.05	6.66
Vanilla Flavor	-	2.03	-	-	-	-
Salt	3.00	2.03	3.00	1.63	-	-
Baking Powder	6.00	0.55	5.50	6.53	9.09	-
Fats in Batter	6%	11%	10%	20%	19%	25%
References	[35]	[89]	[31]	[92]	[93]	[94]

Notes: * White cakes use egg white (not yolk) and shortening, instead of butter.

**Table 5:** Successful fat replacer in bakery products.

Figure	Bakery Products (Flour 100 g)	Replacement % of Full-Fat Bakery Products	Results	References
Beeswax–sunflower oil oleogels	AACC 10–90 cake	100%	SFA 58%→15.5%	[98]
Candelilla wax–canola oil oleogels	AACC 10–54 cookie	30–40%	SFA 63.4%→32.3%	[50]
Carnauba wax–canola oil oleogels	AACC 10–90 cake	25%	SFA 74.2%→64.24%	[99]
Candelilla wax–canola oil oleogels	AACC 10–52 cookie	100%	SFA 52.8%→8.5%	[100]
Inulin from chicory roots.	Sponge cake: 100% sugar, 46% sunflower oil	70%	Reduced fat and fortified fiber	[101]
Inulin	Short dough biscuit: 74.1% margarine; 37% sugar	25%	Textural and sensory properties maintained	[102]
Inulin	Short dough biscuit: 30% shortening; 15% sugar	20%	Weakened lubrication of biscuit dough	[103]
Acetylated rice starch	Cookie: 60% sugar, 30% shortening	20%	Native and modified rice starch equally effective	[104]
Inulin	Biscuit: 45% margarine, 26.7% sugar	20%	Biscuit energy density reduced by 580 kJ/kg	[105]
Corn fiber, maltodextrin or lupine extract	Short dough biscuit: 132% margarine, 66% sugar	30%	28.6% fat reduction and 23 g/kg fiber fortification	[106]
Carnauba wax 5%—cotton oil oleogels	AACC 10–90 cake	50%	SFI similar to shortening fats	[107]
Chia seeds mucilage (80.16% carbohydrate, 10.63–10.76% protein)	AACC 10–90 cake and AACC 10–10 bread	50% and 75%	51.6–56.6% fat reduction and protein fortification	[73]
High-oleic sunflower oil and inulin/β-glucan/lecithin	Biscuit: 34% sugar, 46% shortening	100%	Lecithin (3%, sunflower based) achieved similar sensory quality	[108]
Chia mucilage gel	Pound cake: 75% sugar, 30% shortening	25%	Higher replacement led to adverse effect to color and texture	[109]
Puree of canned green peas	full-fat chocolate bar cookies: 324% sugar, 134% shortening	75%	By sensory assessment	[110]
High oleic sunflower oil + wheat bran (1.9:1)	Cookie: 52% sugar, 33% shortening, 40% egg	100%	SFA: 54.6→24.5%	[111]
77.3:34:12.4:1.3 olive oil: water: inulin: lecithin	Cake: milk fats 35%, sugar 33%, egg 40%	50%	SFA: <39% TL: <19%	[112]

Notes: Solid fat index (SFI); saturated fatty acids (SFA); total lipids (TL).

**Table 6:** Nutritional information of baked goods by US Department of Agriculture (USDA) [2].

Baked Products	Bread	Biscuit	Cookie	Sponge Cake	Pound Cake	Wheat Cracker	White Cake	Yellow Cake
USDA code	18064	21142	3213	18133	45209528	18232	45262644	45174254
Water (g)	35.25	27.88	5.90	29.70	26.25	2.75	24.69	18.83
Energy (kJ)	1145	1547	1812	1213	1516	1903	1653.865	1725.044
Protein (g)	10.67	7.08	11.80	5.4	3.75	7.3	2.47	2.35
Total lipids (g)	4.53	18.92	13.20	2.7	15.00	16.4	18.52	16.47
Ash (g)	2.01	3.31	2.00	1.2	-	2.83	-	-
Carbohydrate (g)	47.54	42.82	67.10	61	55.00	70.73	54.32	62.35
Sugar (g)	5.73	3.88	24.2	36.66	40.00	6.9	-	-
Total dietary fiber (g)	4.00	2.50	0.20	0.5	-	15.48	43.21	49.41
Total saturated FA (g)	0.70	11.80	2.35	0.80	3.75	3.21	7.41	8.24
Total monounsaturated FA (g)	0.61	2.49	5.99	0.95	0.00	3.47	-	-
Total polyunsaturated FA (g)	1.62	2.20	2.88	0.45	0.00	8.474	-	-
Total trans FA (g)	0.03	0.21	0.02	-	3.75	0.034	-	-
